# Efficacy of thyme oil and nano-formulated derivatives against *Rhipicephalus sanguineus* sensu lato (Acari: Ixodidae)

**DOI:** 10.1038/s41598-026-37451-9

**Published:** 2026-02-18

**Authors:** Eman A. Abo Talep, Mai Abuowarda, Sobhy Abdel-Shafy, Fathalla Ayoob, Hoda S. M. Abdel-Ghany, Eman I. Hassanen, Magdy M. Fahmy

**Affiliations:** 1 Department of Parasitology, Faculty of Veterinary Medicine, Cairo University, PO 12211, Giza, Egypt; 2 Department of Parasitology and Animal Diseases, National Research Centre, Veterinary Research Institute, Dokki, PO 12622, Giza, Egypt; 3 Department of Training Materials and Leather Technology, National Research Centre, Chemical Industries Research Institute, Dokki, PO 12622, Giza, Egypt; 4 Department of Pathology, Faculty of Veterinary Medicine, Cairo University, PO 12211, Giza, Egypt

**Keywords:** *Rhipicephalus sanguineus*, Acaricidal activity, Thyme oil, Nano-formulations, Control, Biological techniques, Biotechnology, Nanoscience and technology, Zoology

## Abstract

The brown dog tick, *Rhipicephalus sanguineus* plays a significant role in transmitting pathogens to humans and animals, necessitating control measures. Given the drawbacks of synthetic chemicals, plant-based alternatives are a promising option for tick management. The present study aimed to evaluate the acaricidal potential of thyme oil (TO) and nano-formulations {thyme oil nano-emulsion (TNE), silver nanoparticles (AgNPs), and thyme nano-emulsion containing silver nanoparticles (TNE- AgNPs)} against *R. sanguineus* using an adult immersion bioassay. Gas chromatography-mass spectrometry (GC-MS) was used for TO analysis. The UV- visible spectroscopy, transmission electron microscopy (TEM), and particle size were used to characterize the nano-formulations. In addition, the freshly dead ticks were examined using scanning electron microscopy (SEM). The GC-MS analysis indicated that Thymol (34.08%) was the main oil component, followed by γ-Terpinene (32.99%). The TEM images revealed a spherical-shaped nano droplet with a size of ≤ 100 nm for all nanoformulations. The droplet size and polydispersity index were (445.9 & 0.325), (10.59 & 0.216), and (768.2 & 0.325) for TNE, AgNPs, and TNE-AgNPs, respectively. The calculated LC_50_ values after 7 days were 11.62, 5.47, 4.08, and 2.38% for TO, TNE, AgNPs, and TNE- AgNPs, respectively. The SEM examination revealed changes in the sensilla base, spiracular plate and anal groove region of the treated ticks. Although the biological parameters of the engorged females did not differ significantly between the treatment and control groups, there was a decrease in the hatchability percent of TO (67%), TNE (65%), and TNE-AgNPs (71%) compared to the control (80%). The used materials demonstrated acaricidal activity and might be candidates for managing the dog tick, *R. sanguineus.* Further detailed studies are needed to enable good judgment of the use of these materials to control *R. sanguineus* tick.

**Supplementary Information:**

The online version contains supplementary material available at 10.1038/s41598-026-37451-9.

## Introduction

Ticks and tick-borne pathogens (TBP) pose a significant threat to the livestock industry, causing significant economic losses due to blood loss, disease transmission, and the costs associated with treatment and control measures^1^. *Rhipicephalus sanguineus* (Acari: Ixodidae) are widely distributed geographically and are known to transmit *Rickettsia conorii*, the etiological agent of Boutonneuse Fever in the Mediterranean region^2,3^. In the USA, it has been linked to the transmission of *Rickettsia rickettsii*, causing Rocky Mountain spotted fever^4^. In Egypt, the prevalence rate of tick-borne pathogens among dogs was 23.56%, comprising *Ehrlichia* and *Anaplasma* (Anaplasmataceae) at 11.1% and *Babesia canis* (Piroplasmida; Babesiidae) at 8.2%, which were probably transmitted by *R. sanguineus*^5,6^.

The long-standing reliance on synthetic acaricides for tick control has led to challenges such as resistance, environmental contamination, and toxicity concerns. For instance, *R. sanguineus* has shown resistance to multiple acaricide classes, including amidines, pyrethroids, macrocyclic lactones, and phenylpyrazoles^7–10^. In response, research is increasingly focused on developing a more sustainable and safer option. Plant-based products offer a valuable alternative for tick control. Indeed, various plants and their derivatives have shown acaricidal effects against different tick species, including *Ixodes scapularis*, *Rhipicephalus microplus*, and *Rhipicephalus annulatus*^11–14^. Essential oils, in particular, exhibit a range of activities, including repellent, antifeeding, chemosterilant, and biocidal effects, making them promising candidates for tick management^13,15–17^. With minimal impact on non-target organisms, essential oils are attractive options. These complex mixtures typically contain 20–60 components at varying concentrations^18^. The multi-target action of essential oils’ active compounds may delay resistance development, providing a sustainable solution for tick control^19^.

*Thymus vulgaris*, commonly known as thyme, is an aromatic plant primarily used for flavoring, culinary, and medicinal purposes^20^. It is often extracted as essential oil (EO), and the promising biological activity of thyme EO, including antimicrobial and antioxidant activity, has been demonstrated^21,22^. Thyme EO has also been found to be effective against insect pests^23^ and ticks, including *R. sanguineus*^24^. Thymol, the primary component of thyme EO, has shown acaricidal activity against various tick species, including *Ixodes ricinus*^25^, *R. sanguineus*, *R. microplus*, and *R. annulatus*^26–29^.

Despite the enormous potential of application of thyme EO, several limitations limit the use of plant EOs in pest management, including poor water solubility, high volatility, and rapid degradation^30,31^. Therefore, the development of stable and effective acaricide formulations is necessary. Nanotechnology, including nano-emulsion and nano-encapsulation, is a promising approach for improving the properties of plant EOs^30,31^. Several studies have reported the acaricidal activity of nano-emulsions against *R. microplus* larvae^32^ and *R. microplus* females^33,34^. In addition, Marimuthu et al.^35^ tested the biosynthesized silver nanoparticles from *Mimosa pudica* Gaertn leaf extract against the larvae of *R. microplus.* Silver nanoparticles (AgNPs) synthesized by subjecting myrrh and ginger extracts to laser ablation have been shown to exert potent toxic effects on *Hyalomma dromedarii* ticks, impacting both adult and immature stages^36^. Studies have demonstrated that AgNPs can cause significant mortality through mechanisms involving oxidative stress, membrane disruption, and interference with key physiological processes in ticks^37^. In addition to direct lethality, AgNPs can negatively affect reproductive parameters such as egg laying and hatchability, where^38^ reported that green-synthesized silver nanoparticles using *Andrographis paniculata* extract significantly reduced the survival rate of *R. microplus* ticks and inhibited oviposition in treated females.

The present study aimed to evaluate the acaricidal activity of thyme EO and its nano-formulations against *R. sanguineus*. While a prior study has examined the acaricidal activity of thyme EO and its nano–formulation against *R. sanguineus*^24^, our study expands on this knowledge by providing a comprehensive and in-depth evaluation of their acaricidal activity, including morpho-histological changes.

## Materials and methods

### Ticks

Fully fed female *R. sanguineus* specimens (*N* = 100) were obtained from a well-established colony at Cairo University’s Department of Parasitology of Veterinary Medicine, located in Giza, Egypt. These females were placed in a plastic cup for oviposition which lasted 10 days and maintained in an incubator at 25 °C ± 1 °C and 75%–80% relative humidity. After 16 days of incubation, eggs hatched into larvae. Hungry larvae were fed on healthy rabbits using the capsule technique one week later^39–41^. The detached, fully engorged larvae were collected and incubated for one week under the same conditions to molt into nymphs. Hungry nymphs were reared on healthy rabbits to obtain fully engorged nymphs that were incubated for two weeks to molt into unfed adults. In male ticks, the dorsal shield (scutum) covers most of the body, whereas in female ticks, it only covers a portion of the body^41^.

### Source of oil and GC–MS analysis

Thyme oil (TO) was purchased from Ab Chem Company (https://www.abchem.org**).** Thyme oil analysis was conducted using gas chromatography mass spectrometry (Agilent 8890 GC System, Santa Clara, California), which was joined to an Agilent 5977B GC/MSD mass spectrometer and equipped with an HP-5MS fused silica capillary column (Santa Clara, California, USA) (30 m, 0.25 mm i.d., 0.25 mm film thickness). At first, the temperature was maintained at 50 °C. After that, it automatically climbed by 5 °C/min to 200 °C and fell by 10 °C/min to 280 °C. At last, it was kept for seven minutes at 280 °C. Helium was used as the carrier gas, and its flow rate was 1.0 mL/min. 1 µL of the dissolved essential oil (20 µL essential oil / mL diethyl ether) was inoculated into the gas chromatograph with a split ratio of 1:50. The temperature of the injection. 230 °C was the injection temperature. In the electron impact mode (EI), mass spectra were achieved at 70 eV, covering a range of 39 to 500 amu in m/z. Data from the mass spectra database (National Institute of Standards and Technology, NIST) were compared with the isolated peaks^41^.

### Preparation of thyme oil nano-emulsion (TNE)

A low-energy method was used for the preparation of emulsion according to Sugumar et al.^42^, nano-emulsions were formed with little modification. The oil-in-water nano-emulsion was formed using thyme oil, non-ionic surfactant (Tween 80, MP Biomedicals, United States), and distilled water. In all formulations, the oil concentration (20% v/v) was fixed as a stock solution. The ratio of non-ionic surfactant (Tween 80) to oil was (1:1) at room temperature. After that, the mixture was homogenized, and then the distilled water was added intermittently during stirring using a magnetic stirrer at a high speed of 2000 rpm for 3 h. Finally, the obtained nano-emulsion was left under stirring overnight.

### Preparation of thyme nano-emulsion containing silver nanoparticles (TNE- AgNPs)

Silver nanoparticles were purchased from the Nano Gate Company (https://nanogate-eg.com/en). Thyme nanoemulsion mixed with silver nanoparticles (TNE-AgNPs) was prepared by adding 5% AgNPs to 20% thyme nanoemulsion. The AgNPs were synthesized by introducing 0.1 mM AgNO3 to 10 mL of water, adjusting the pH to 10, and heating the solution for 60 min while stirring until it turned dark yellowish-brown. Equal amounts of the AgNPs solution were then combined with the thyme nanoemulsion to produce TNE-AgNPs.

### Characterization of prepared nano-formulations

#### UV-visible spectroscopy

The characterization of TNE and TNE - AgNPs was performed by spectrophotometric analysis (T80 UV/VIS Spectrometer) through a scanning wavelength range of 200–900 nm. The UV–Vis spectral determination gives insight into the actual formation of the nano-formulations by surface plasmon resonance effect^43^.

#### Transmission electron microscopy (TEM)

The transmission electron microscope was used to measure the sample droplet size. A few drops of the sample and 1% phosphotungstic acid were placed on a carbon-coated copper grid and allowed to dry after the suspension of the materials was sonicated for 20 min on an ultrasonicator (Crest Ultrasonics Corp., New Jersey, USA). Next, the sample-loaded grid was subjected to an examination by a 200 kv HR-TEM (JEOL, JEM-2100, Tokyo, Japan)^42,43^.

#### Particle size

Using the dynamic light scattering (DLS) method and a Malvern Zetasizer 3000 HAS (Malvern Instruments, Ltd., UK), the particle size distribution was analyzed at the following conditions: temperature of 23 °C, run time of 2 min, solvent of water, and concentration of 1 mg/ml^43^.

#### Tick bioassay using unfed adult immersion test (AIT)

Unfed adult ticks were immersed at different concentrations of TO (40, 30, 20, 10, 5%), TNE (30, 20, 10, 5, 2.5%), AgNPs, and TNE - Ag NPs (5, 4, 3, 2, 1%), which were chosen according to pilot studies. A total of 96 replicates were distributed as three replicates per treatment/tested material (10 ticks/ replicate, five males and five females) of unfed adults were used for each concentration, and the immersion time was 5 min in 2 ml/concentration. Furthermore, unfed adults were immersed in the following solutions: 5% Tween 80 as solvent control (control with Tween), Butox^®^ 5.0 (Deltamethrin) 1 ml/L as a recommended concentration of acaricidal reference (10 µl Butox^®^ 5.0 diluted in 10 ml water), and distilled water only as negative control (control without Tween). Treated ticks were incubated in glass tubes at 25 °C ± 1 °C and 75%–80% relative humidity. Mortality rates were assessed over seven consecutive days to capture any delayed effects, with ticks considered dead if they failed to respond to exhalation-induced stimulation^42,43^.

#### Scanning electron microscopy for tick specimens

The five unfed adult tick specimens (3 females and 2 males) treated with 40% TO, 30% TNE, 5% AgNPs, and 5% TNE-AgNPs for 5–7 days, as well as control ticks treated with distilled water were well cleaned by immersion in water–glycerol– potassium chloride (KCl) solution overnight^44,45^. The ticks were then washed in distilled water several times, followed by immersion in a series of ethanol concentrations: 25%, 50%, 70%, and 80% for 1 h each, and then 90% and 100% ethanol for 10 min each^46^. Ethanol dehydration was employed to preserve tick samples, as it effectively replaces water without inducing structural collapse or distortion, thereby maintaining morphology for high-quality scanning electron microscopy imaging. Ticks were then adhered to the SEM stub by their dorsal and ventral surfaces, and liquid carbon dioxide was used to dry them in a drier (Blazer Union, F1-9496 Blazer/Furstentum Liechtenstein). A S15OA Sputter Coater was used to apply gold coating to ticks that were fixed on SEM stubs (Ted Pella, Inc., Redding, California, United States). The SEM investigation of the coated samples was performed at the National Research Centre using SEM (Quanta FEG 250).

#### Effect of 50% lethal concentration (LC_50_) on the reproductive performance of tick females

This experiment aimed to examine the impact of TO, TNE, AgNPs, and TNE-AgNPs on the progeny of unfed adult ticks that survived exposure to LC_50_ concentrations and later fed on rabbits. The unfed adult of *R. sanguineus* ticks (20 males and 20 females/conc) were immersed for five minutes at concentrations of the calculated LC_50_ after 7 days (11.62, 5.47, 4.08, and 2.38%) for TO, TNE, AgNPs, and TNE - AgNPs, respectively. After 24 h, surviving ticks were reared on rabbits using the capsule technique (3 rabbits /conc) to follow the reproductive performance of tick females. Following engorgement, reproductive parameters were calculated for engorged females, including pre-oviposition weight, egg mass weight, egg production index (EPI), oviposition duration, egg incubation period, and hatchability percentage, as per established methods Abo Talep et al.^39^. The oviposition period was determined by the average time from starting to end laying eggs. The incubation period of eggs was estimated by the average time from start laying eggs to start hatching larvae. The egg productive index (EPI) and hatchability of the laid eggs were calculated as follows: EPI = egg mass weight (mg)/initial weight of engorged females (mg)^47^. Percentage of hatchability = Number of hatched larvae/number of laid eggs × 100^48^. Tick eggs were counted before hatching by weighing the egg mass per female, taking a portion, weighing it, and counting it. The total number of eggs per egg mass was then calculated. Larvae were counted for each female after hatching was complete in the untreated group.

### Statistical analysis

The percent mortality of unfed adults treated with TO, TNE, Ag NPs, and TNE – AgNPs was statistically analyzed by a one-way ANOVA test followed by Tukey’s test.

using SPSS program version 20. By using regression equation analysis on the probit-transformed mortality data, 50% lethal concentration (LC_50_) values were determined using Ehab software. The probit approach was used to examine the dose-response data^49^.

## Results

### Gas chromatography – mass spectrometry (GC–MS) analysis

Ten components were identified in thyme oil using the GC-MS library (Table 1). The analysis indicated that Thymol (34.08%) was the main oil component and followed by γ-Terpinene (32.99%).

**Table 1:** Chemical compositions of thyme oil.

Component name	Area	Area sum (%)	RT	Peak
β-Thujene	958475.45	0.31	6.166	1
α-Pinene	2073423.31	0.67	6.339	2
β-Pinene	11339346.81	3.67	7.396	3
β-Myrcene	1708838.97	0.55	7.714	4
α-Terpinene	1313769.11	0.42	8.413	5
p-Cymene	80688018.68	26.08	8.65	6
D-Limonene	2839724.36	0.92	8.754	7
γ-Terpinene	102075747.2	32.99	9.591	8
Cosmen-2-ol	942248.65	0.3	12.565	9
Thymol	105452858.3	34.08	16.088	10

RT: Retention time in seconds.

### Characterization of prepared nano-formulations

#### UV-visible spectroscopy

The absorption peak maxima of TNE were observed at 220 nm. In contrast the absorption peak maxima of TNE- AgNPs showed at 225 nm. (Fig. S1).

#### Transmission electron microscopy (TEM)

**Fig. 1:**
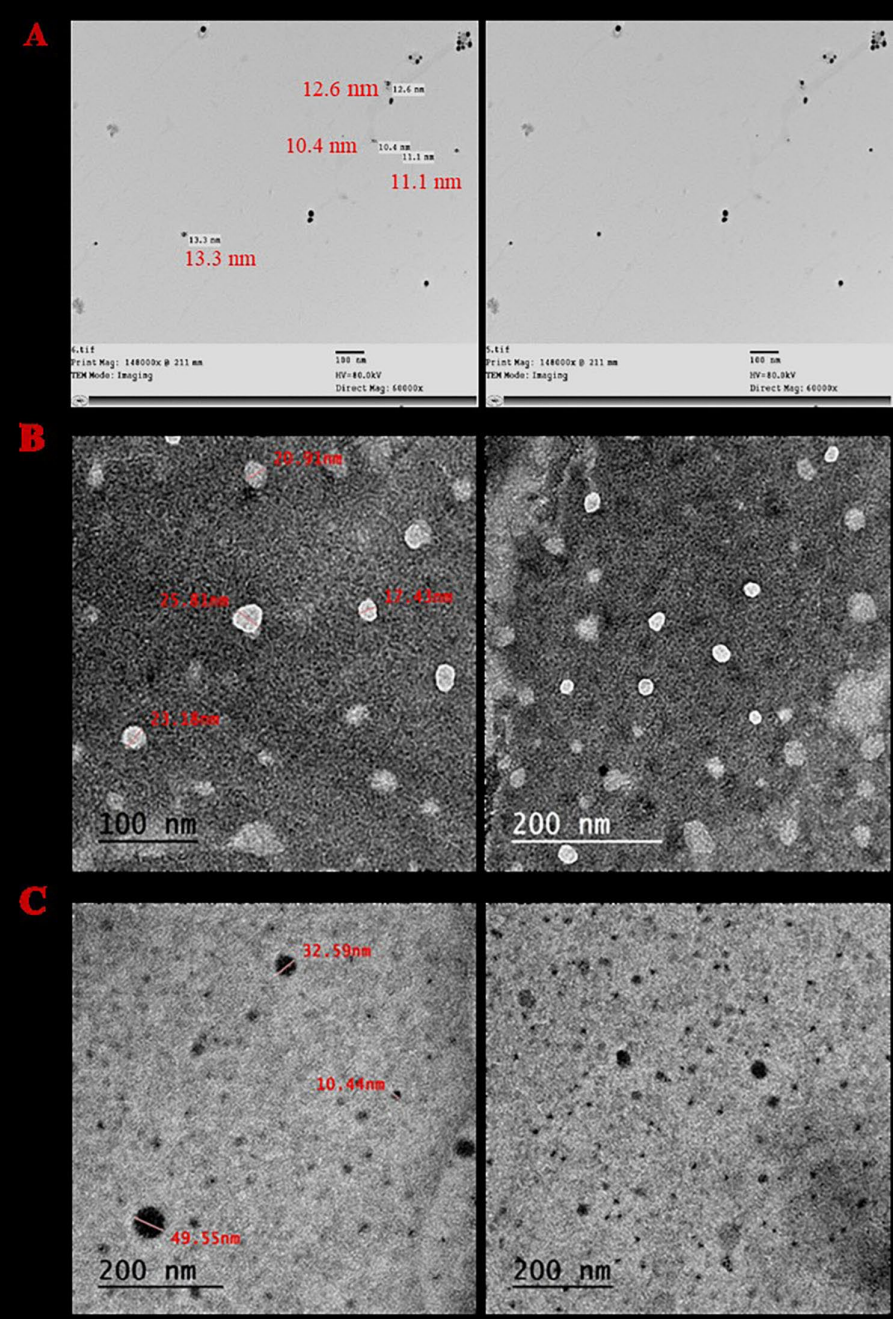
Transmission electron micrographs: (**A**) silver nanoparticles (AgNPs), (**B**) thyme nano-emulsions (TNE), (**C**) thyme nano-emulsion containing silver nanoparticles (TNE - AgNPs).

The results of TEM indicated that TNE droplets, AgNPs, and TNE-AgNPs appeared spherical without aggregations, and the size within the range of nanoparticles (≤ 100 nm) where the size ranged from (10–48), (11–14.7 nm), and (10.44–49.55), respectively (Fig. 1).

#### Particle size

The particle size for TNE, AgNPs, and TNE-AgNPs were 445.9, 10.59, and 768.2 nm, with a correspondingly low PDI of 0.325, 0.216, and 0.320, respectively (Table 2 and Fig. S2).

**Table 2:** Particle size of thyme nano-emulsion (TNE) and thyme nano-emulsion contaning silver nanoparticles (TNE-AgNPs) and silver nanoparticles (AgNPs).

Sample	Particle size (nm)	Polydispersity index (PDI)
TNE	445.9	0.325
TNE – AgNPs	768.2	0.320
AgNPs	10.59	0.216

### Acaricidal effect of TO, TNE, ag NPs, and TNE-Ag NPs

The efficacy of thyme oil (TO) and its nano-formulation (TNE) against unfed *R. sanguineus* adults is presented in Tables 3 and 4. Mortality rates increased with both time and concentration. At 40%, TO achieved 93% mortality on day 7, while TNE reached 100% mortality on day 6. Even at the lowest concentration (5%), TO induced mortality on day 5, whereas TNE showed effects at 2.5% on day 3. These findings indicate TNE’s slightly superior efficacy. Notably, both TO and TNE showed no significant difference in effectiveness compared to the acaricide Butox at the highest concentration (*P* ≥ 0.05).

**Table 3:** Mortality rate of unfed adult ticks *R. sanguineus* treated with different concentrations of thyme oil (TO) for 7 successive days.

Treatments	Mortality (%)						
	1st day	2nd day	3rd day	4th day	5th day	6th day	7th day
40% oil	30.00 ± 10.00^ab^	50.00 ± 10.00^c^	56.67 ± 13.33^c^	60.00 ± 15.28^b^	66.67 ± 8.82^d^	76.67 ± 6.67^c^	93.33 ± 6.67^ef^
30% oil	3.3 ± 3.33^a^	10.00 ± 5.77^a^	23.33 ± 6.67^ab^	33.33 ± 3.33^ab^	56.67 ± 3.33c^d^	66.67 ± 3.33^c^	76.67 ± 3.33^de^
20% oil	0.00 ± 0.00^a^	0.00 ± 0.00^ab^	13.33 ± 3.33^ab^	16.67 ± 6.67^a^	33.33 ± 3.33b^c^	46.67 ± 3.33^b^	60.00 ± 5.77^cd^
10% oil	0.00 ± 0.00^a^	0.00 ± 0.00^a^	0.00 ± 0.00^a^	3.33 ± 3.33^a^	26.67 ± 6.67^abc^	43.33 ± 3.33^b^	53.33 ± 3.33^c^
5% oil	0.00 ± 0.00^a^	0.00 ± 0.00^a^	0.00 ± 0.00^a^	0.00 ± 0.00^a^	3.33 ± 3.33^ab^	13.33 ± 3.33^a^	20.00 ± 5.77^b^
Butox^®^	20.00 ± 5.77^b^	23.33 ± 3.33^b^	30.00 ± 5.77^bc^	60.00 ± 10.00^b^	76.67 ± 13.33^d^	93.33 ± 6.67^d^	100.00 ± 0.00^f^
Control without Tween	0.00 ± 0.00^a^	0.00 ± 0.00^a^	0.00 ± 0.00^a^	0.00 ± 0.00^a^	0.00 ± 0.00^a^	0.00 ± 0.00^a^	0.00 ± 0.00^a^
Control with Tween	0.00 ± 0.00^a^	0.00 ± 0.00^a^	0.00 ± 0.00^a^	0.00 ± 0.00^a^	0.00 ± 0.00^a^	0.00 ± 0.00^a^	0.00 ± 0.00^a^
F(7,16)	7.560	18.011	12.565	13.841	22.967	75.571	95.131
P	< 0.001	< 0.001	< 0.001	< 0.001	< 0.001	< 0.001	< 0.001

Means within a column and followed by different letters are significantly different (Tukey test: *P* < 0.05).

**Table 4:** Mortality rate of *R. sanguineus* unfed adult ticks treated with different concentrations of thyme nano-emulsion (TNE) for 7 successive days. Means within a column and followed by different letters are significantly different (Tukey test: *P* < 0.05).

Treatments	Mortality (%)						
	1st day	2nd day	3rd day	4th day	5th day	6th day	7th day
30% TNE	30.00 ± 5.77^b^	50.00 ± 5.77^c^	56.67 ± 8.82^c^	80.00 ± 15.28^c^	93.33 ± 6.67^c^	100.00 ± 0.00^e^	100.00 ± 0.00^d^
20% TNE	3.33 ± 3.33^a^	10.00 ± 0.00^a^	20.00 ± 10.00^ab^	26.67 ± 16.67^ab^	80.00 ± 10.00^c^	86.67 ± 3.33^de^	93.33 ± 3.33^d^
10% TNE	0.00 ± 0.00^a^	10.00 ± 0.00^a^	13.33 ± 3.33^ab^	23.33 ± 8.82^ab^	50.00 ± 15.28^bc^	63.33 ± 14.53^cd^	70.00 ± 15.28^cd^
5% TNE	0.00 ± 0.00^a^	10.00 ± 0.00^a^	10.00 ± 0.00^ab^	10.00 ± 0.00^a^	20.00 ± 10.00^ab^	33.33 ± 3.33^bc^	46.67 ± 6.67^bc^
2.5% TNE	0.00 ± 0.00^a^	0.00 ± 0.00^a^	3.33 ± 3.33^a^	10.00 ± 0.00^a^	13.33 ± 3.33^ab^	20.00 ± 5.77^ab^	20.00 ± 5.77^ab^
Butox^®^	20.00 ± 5.77^b^	23.33 ± 3.33^b^	30.00 ± 5.77^b^	60.00 ± 10.00^bc^	76.67 ± 13.33^c^	93.33 ± 6.67^de^	100.00 ± 0.00^d^
Control without Tween	0.00 ± 0.00^a^	0.00 ± 0.00^a^	0.00 ± 0.00^a^	0.00 ± 0.00^a^	0.00 ± 0.00^a^	0.00 ± 0.00^a^	0.00 ± 0.00^a^
Control with Tween	0.00 ± 0.00^a^	0.00 ± 0.00^a^	0.00 ± 0.00^a^	0.00 ± 0.00^a^	0.00 ± 0.00^a^	0.00 ± 0.00^a^	0.00 ± 0.00^a^
F(7,16)	14.041	51.679	12.626	9.859	17.448	44.362	46.379
P	< 0.001	< 0.001	< 0.001	< 0.001	< 0.001	< 0.001	< 0.001

Concerning AgNPs and TNE-AgNPs, the mortalities on the 7th day were 80% and 96.6% at the highest concentration of 5%, respectively. At the concentration of 1% AgNPs recorded no mortality while TNE-AgNPs recorded 13.3% mortality. No significant difference (*P* ≥ 0.05) was presented between TNE-Ag NPs and Butox^®^ (Table 5). While there was significance between AgNPs and Butox^®^ at the highest concentration (Table 6). There were no mortalities recorded in the controls. The calculated LC_50_ value after 7 days was 11.62, 5.47, 4.08, and 2.38 for TO, TNE, AgNPs, and TNE-Ag NPs, respectively (Table 7). From the calculated LC_50_, TNE-AgNPs were more toxic against unfed adults of *R. sanguineus*, followed by AgNPs, TNE, and TO.

**Table 5:** Mortality rate of *R. sanguineus* unfed adult treated with different concentrations of thyme nano-emulsion containing silver nanoparticles (TNE -Ag NPs) for 7 successive days.

Treatments	Mortality (%)						
	1st day	2nd day	3rd day	4th day	5th day	6th day	7th day
5% TNE -Ag NPs	0.00 ± 0.00^a^	10.00 ± 10.00^ab^	43.33 ± 6.67^c^	73.33 ± 3.33^c^	90.00 ± 0.00^c^	93.33 ± 3.33^d^	96.67 ± 3.33^d^
4% TNE -Ag NPs	0.00 ± 0.00^a^	3.33 ± 3.33^ab^	16.67 ± 3.33^ab^	30.00 ± 5.77^b^	40.00 ± 5.77^b^	63.33 ± 8.82^c^	73.33 ± 13.33^c^
3% TNE -Ag NPs	0.00 ± 0.00^a^	3.33 ± 3.33^ab^	6.67 ± 6.67^a^	10.00 ± 5.77^ab^	13.33 ± 6.67^ab^	26.67 ± 8.82^b^	60.00 ± 5.77^c^
2% TNE -Ag NPs	0.00 ± 0.00^a^	3.33 ± 3.33^ab^	6.67 ± 6.67^a^	6.67 ± 6.67^ab^	10.00 ± 5.77^a^	13.33 ± 3.33^ab^	33.33 ± 3.33^b^
1% TNE -Ag NPs	0.00 ± 0.00^a^	0.00 ± 0.00^a^	0.00 ± 0.00^a^	0.00 ± 0.00^a^	3.33 ± 3.33^a^	6.67 ± 3.33^a^	13.33 ± 8.82^a^
Butox^®^	20.00 ± 5.77^b^	23.33 ± 3.33^b^	30.00 ± 5.77^bc^	60.00 ± 10.00^c^	76.67 ± 13.33^c^	93.33 ± 6.67^d^	100.00 ± 0.00^d^
Control without Tween	0.00 ± 0.00^a^	0.00 ± 0.00^a^	0.00 ± 0.00^a^	0.00 ± 0.00^a^	0.00 ± 0.00^a^	0.00 ± 0.00^a^	0.00 ± 0.00^a^
Control with Tween	0.00 ± 0.00^a^	0.00 ± 0.00^a^	0.00 ± 0.00^a^	0.00 ± 0.00^a^	0.00 ± 0.00^a^	0.00 ± 0.00^a^	0.00 ± 0.00^a^
F(7,16)	12.000	3.505	11.634	30.714	34.519	55.667	43.628
P	< 0.001	0.018	< 0.001	< 0.001	< 0.001	< 0.001	< 0.001

Means within a column and followed by different letters are significantly different (Tukey test: *P* < 0.05).

**Table 6:** Mortality rate of *R. sanguineus* unfed adult treated with different concentrations of silver nanoparticles (AgNPs) for 7 successive days.

Treatments	Mortality (%)						
	1st day	2nd day	3rd day	4th day	5th day	6th day	7th day
5% Ag NPs	3.33 ± 3.33^a^	10.00 ± 5.77^a^	13.33 ± 3.33^b^	23.33 ± 3.33^a^	43.33 ± 0.00^b^	53.33 ± 8.82^b^	80.00 ± 5.77^d^
4% Ag NPs	0.00 ± 0.00^a^	3.33 ± 3.33^a^	3.33 ± 3.33^ab^	13.33 ± 8.82^a^	30.00 ± 5.77^b^	33.33 ± 8.82^b^	40.00 ± 5.77^c^
3% Ag NPs	0.00 ± 0.00^a^	0.00 ± 0.00^a^	0.00 ± 0.00^a^	0.00 ± 0.00^a^	3.33 ± 3.33^a^	6.67 ± 3.33^a^	16.67 ± 3.33^b^
2% Ag NPs	0.00 ± 0.00^a^	0.00 ± 0.00^a^	0.00 ± 0.00^a^	0.00 ± 0.00^a^	0.00 ± 0.00^a^	0.00 ± 0.00^a^	0.00 ± 0.00^a^
1% Ag NPs	0.00 ± 0.00^a^	0.00 ± 0.00^a^	0.00 ± 0.00^a^	0.00 ± 0.00^a^	0.00 ± 0.00^a^	0.00 ± 0.00^a^	0.00 ± 0.00^a^
Butox^®^	20.00 ± 5.77^b^	23.33 ± 3.33^b^	30.00 ± 5.77^c^	60.00 ± 10.00^b^	76.67 ± 13.33^c^	93.33 ± 6.67^c^	100.00 ± 0.00^e^
Control without Tween	0.00 ± 0.00^a^	0.00 ± 0.00^a^	0.00 ± 0.00^a^	0.00 ± 0.00^a^	0.00 ± 0.00^a^	0.00 ± 0.00^a^	0.00 ± 0.00^a^
Control with Tween	0.00 ± 0.00^a^	0.00 ± 0.00^a^	0.00 ± 0.00^a^	0.00 ± 0.00^a^	0.00 ± 0.00^a^	0.00 ± 0.00^a^	0.00 ± 0.00^a^
F(7,16)	8.821	10.029	16.800	19.084	28.054	45.233	165.694
P	< 0.001	< 0.001	< 0.001	< 0.001	< 0.001	< 0.001	< 0.001

Means within a column and followed by different letters are significantly different (Tukey test: *P* < 0.05).

**Table 7:** LC_50_ and LC_99_ values with their confidence limits for *R. sanguineus* unfed adult treated with thyme oil (TO), thyme nano-emulsion (TNE), thyme nano-emulsion containing silver nanoparticles (TNE- AgNPs), and silver nanoparticles (AgNPs), which were calculated after 7 days.

Treatments	LC_50_ (%)	LC_99_ (%)	Confidence limits				Slope ± SE
			LC_50_ ( % )		LC_99_ ( % )		
			Lower	Upper	Lower	Upper	
TO	11.62	146.96	5.84	17.21	151.76	1493.45	2.11 ± 0.196
TNE	5.47	40.33	4.81	6.16	31.28	56.39	2.68 ± 0.206
TNE- Ag NPs	2.38	10.63	1.57	3.18	11.01	39.97	3.58 ± 0.297
Ag NPs	4.08	7.913	3.90	4.28	6.93	9.73	8.1 ± 0.938

LC_50_: Lethal concentration for 50% of individuals, LC_99_: Lethal concentration for 99% of individuals.

### Scanning electron microscopy (SEM)

**Fig. 2:**
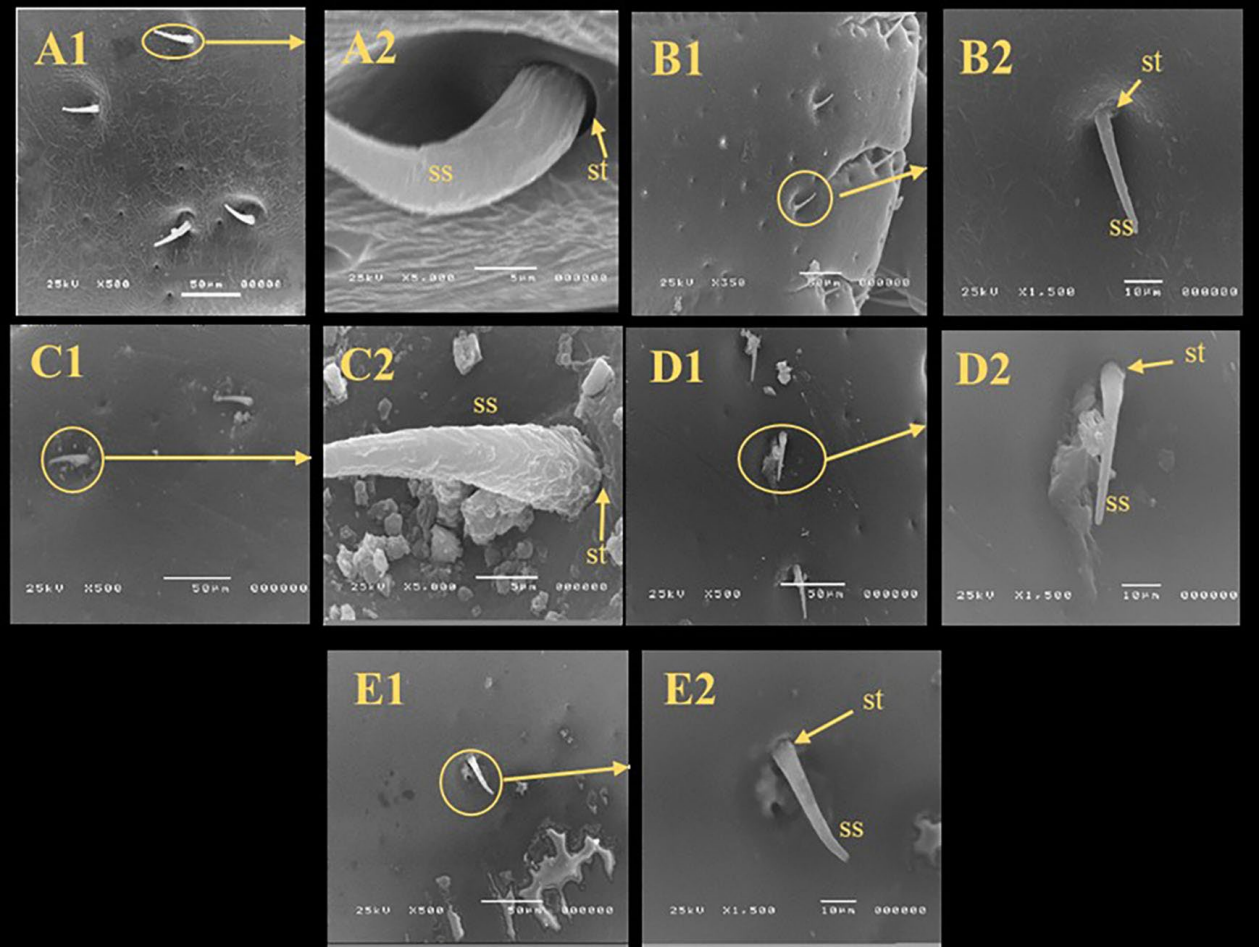
Scanning electron microscopy of sensile and the sensile socket of unfed adult males *R. sanguineus* with different treatments. (**A**) Untreated Control, (**B**) Butox^®^, (**C**) thyme oil (TO), (**D**) thyme nano-emulsion (TNE), (**E**) thyme nano-emulsion containing silver nanoparticles (TNE-AgNPs), **ss** (sensile), st (sensile socket).

**Fig. 3:**
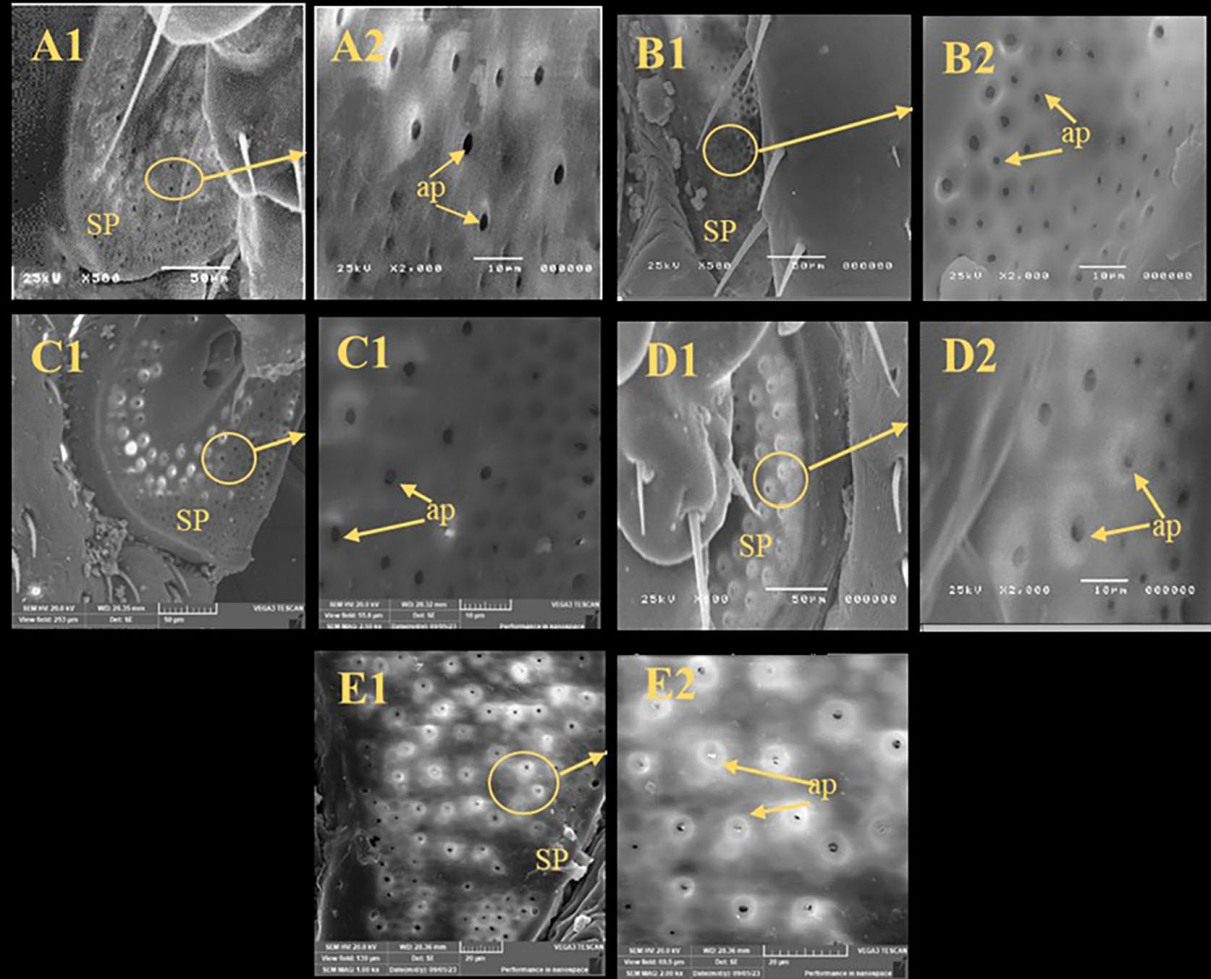
Scanning electron microscopy of the spiracular plate of unfed adult males *R. sanguineus* with different treatments. (**A**) Untreated Control, (**B**) Butox^®^, (**C**) thyme oil (TO), (**D**) thyme nano-emulsion (TNE), (**E**) thyme nano-emulsion containing silver nanoparticles (TNE-AgNPs), **SP** (spiracular plate), **ap** (aeropyle).

**Fig. 4:**
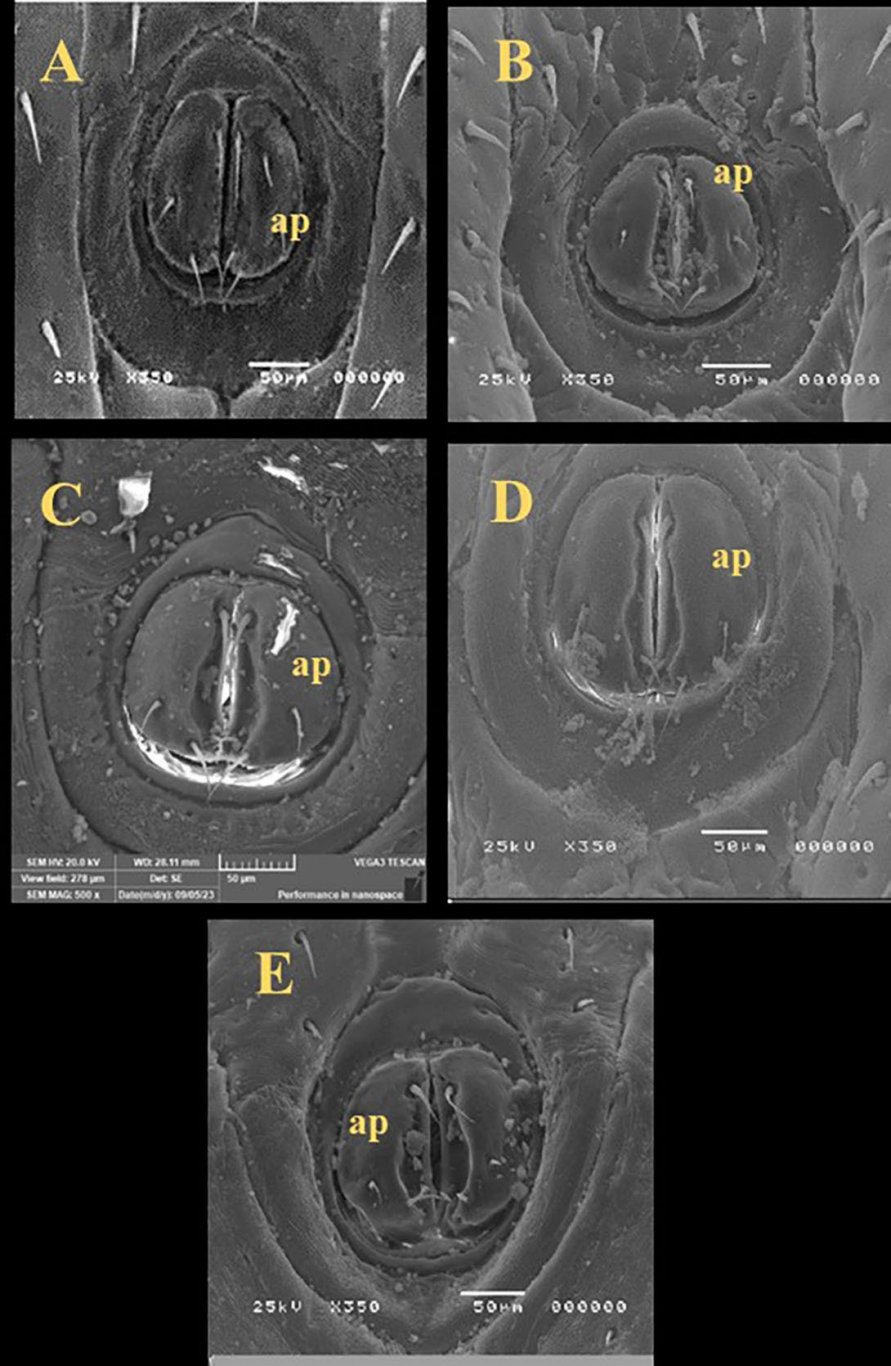
Scanning electron microscopy of the anal opening of unfed adult males *R. sanguineus* with different treatments. (**A**) Untreated (Control), (**B**) Butox, (**C**) thyme oil (TO), (**D**) thyme nano-emulsion (TNE), (**E**) thyme nano-emulsion containing silver nanoparticles (TNE-AgNPs), **ap** (anal opening).

There were irregular spots on the sensilla base of the all treated ticks while the control group was smooth and integrated in the socket (Fig. 2). In comparing with control, there were changes observed in the spiracular plate of all treated groups in which some aeropyles were obstructed by secretions however the aeropyles of control group were clear and distinct (Fig. 3). The anal groove region showed cuticular disorders and filled with irregular spots (Fig. 4).

### Effect of lethal concentration of 50% ticks (LC_50_) on the reproductive performance of tick females

The biological parameters of engorged females, including female weight, egg weight and number, reproductive index (REPI), oviposition, incubation periods, and hatching percent, are shown in Table 8. Engorged female weights weren’t significantly different across treatments (0.18–0.22 g). However, egg mass weights (0.07–0.12 g), REPI (0.39–0.55), and egg numbers varied significantly (TO: 1552 eggs, AgNPs: 1641 eggs, TNE: 1915 eggs, Control: 2019 eggs, TNE-AgNPs: 2533 eggs). Oviposition period was similar (14.69–15.29 days). TO-exposed ticks had shorter incubation period (18.8 days) vs. other treatments (19.27-20 days).There was a decrease in the hatchability percent of TO (67%), TNE (65%), and TNE-AgNPs (71%) than the control (80%).

**Table 8:** The biological parameters of engorged females of *R. sanguineus* treated with LC_50_ concentrations of thyme oil (TO), thyme nano-emulsion (TNE), thyme nano-emulsion containing silver nanoparticles (TNE- AgNPs), and silver nanoparticles (AgNPs).

Treatments	Biological parameters						
	Engorged female weight (g)	Weight of egg mass (g)	REPI	Number of egg	Oviposition period (day)	Incubation period (day)	Hatching percentage (%)
Control	0.19 ± 0.01^a^	0.096 ± 0.008^ab^	0.50 ± 0.034^ab^	2,019±160^ab^	15.29 ± 0.34^a^	19.88 ± 0.12^b^	80.71 ± 7.59^a^
TO	0.19 ± 0.018^a^	0.07 ± 0.006^a^	0.39 ± 0.01^a^	1,552±133^a^	15.20 ± 0.37^a^	18.80 ± 0.49^a^	67.50 ± 15.03^a^
TNE	0.21 ± 0.012^a^	0.09 ± 0.008^ab^	0.44 ± 0.04^ab^	1,915 ± 178^ab^	14.55 ± 0.21^a^	19.27 ± 0.30^ab^	65.29 ± 12.28^a^
TNE-AgNPs	0.22 ± 0.009^a^	0.12 ± 0.005^b^	0.55 ± 0.01^b^	2,533 ± 109^b^	15.63 ± 0.39^a^	20.00 ± 0.09^b^	71.38 ± 9.63^a^
AgNPs	0.18 ± 0.009^a^	0.08 ± 0.008^a^	0.43 ± 0.037^ab^	1,641±177^a^	14.69 ± 0.49^a^	19.69 ± 0.25^ab^	85.97 ± 4.55^a^
F(4,60)	2.557	5.456	3.328	5.456	1.233	3.329	0.921
P value	0.050	0.001	0.016	0.001	0.306	0.016	0.458

Means within a column and followed by different letters are significantly different (Tukey test: *P* < 0.05).

REPI: Reproductive index.

## Discussion

The growing resistance of ticks to synthetic acaricides poses significant challenges to effective tick management, highlighting the urgent need for alternative management strategies^50^. In this study, the acaricidal activity of TO and its nanoformulations was investigated against *R. sanguineus*, and the morphological changes in the treated tick were elucidated.

The GC-MS analysis of TO revealed that Thymol was the major constituent, followed by γ-Terpinene. This result is consistent with Alibeigi et al.^24^, who identified Thymol (38.37%) and γ-Terpinene (15.09%) as the major components. On the other hand, the study of de Oliveira et al.^51^ revealed Thymol (40%), p-cymene (19.2%), and γ-terpinene (17.3%) as the most active constituents. The difference in the percentage of each component may be attributed to physiological and environmental conditions, genetic species, harvesting time, geographical location, and extraction methods^52,53^.

The characteristics of the prepared nanoformulations revealed spherical particles within the nanoscale range (≤ 100 nm), consistent with the fact that efficient nano-droplet sizes range from 20 to 200 nm^54^. Moreover, the low PDI values (0.2 to 0.3) suggest overall stability and homogeneity of the nanoformulations.

The current study demonstrated that TO and its nano-formulated derivatives had acaricidal activity against *R. sanguineus*. However, the observed acaricidal activity varied based on the treatment type and exposure period. Moreover, we observed that TNE-AgNPs, followed by AgNPs, and TNE had higher acaricidal activity against the unfed adult ticks than TO, as indicated by their LC_50_ values. These findings are similar to a previous study, which found that *T. vulgaris* nano EO had higher acaricidal activity (LC_50_ = 0.09%) against *R. sanguineus* compared to *T. vulgaris* EO (LC_50_ = 0.69%)^24^. Besides, enhanced biological activity of plant EOs when prepared and tested as nanoformulations has also been reported for other arthropod pests. For instance, it was reported that *Schinus terebinthifolius* nanoemulsion demonstrated enhanced larvicidal (LC_50_ = 6.8 µl L-1) and adulticidal (LC_50_ = 5.3 µl L-1) activity against *Culex pipiens* compared to the crude EO, which had LC_50_ values of 11.3 and 9.1 µl L-1, respectively^55^. The distinct physical and morphological characteristics of the nanoformulations may have influenced their acaricidal activity against the tick. Indeed, the 5 subcellular size and high specific surface area of nanopesticides (nanoemulsions) enhance their affinity for targeted biological system, improving their spread, bio-membrane permeability, and uptake, thereby enhancing their biological activities^55–57^. While the inherent properties of the nanoformulations may have enhanced their acaricidal activity against *R. sanguineus*, plant EOs are a complex mixture of volatile compounds, which derive their biological activity from these chemical constituents. Thymol found in the TO composition has been reported to have larvicidal activity (0.67–2.12 mg/mL) against *R. microplus* populations from different regions of Brazil^58^. It was also found to have larvicidal activity (LC_50_ = 6.4 mg/mL) against *R. sanguineus*^24^. Moreover, thymol exhibited adulticidal activity (LC_50_ = 1183.9 mg/L) against the carmine spider mites, *Tetranychus cinnabarinus* [Wu et al., 2017]. Furthermore, thymol in combination with other active compounds of TO has been shown to produce synergistic acaricidal activity against *T. cinnabarinus*^59^. Therefore, it is also possible that the enhanced acaricidal activity of the nanoformulations against *R. sanguineus* may be due to the way the bioactive compounds of TO interact with the carrier in the nanoformulation, resulting in their synergistic interactions or improved bioavailability, stability/solubility, and controlled release^60^.

Although the biological parameters of the engorged females did not differ significantly between the treatment and control groups, we found reduced hatchability of *R. sanguineus* eggs after treatment with TO, TNE, and TNE-AgNPs. A similar effect on the hatchability of ticks after treatment with plant-based acaricides has been reported elsewhere^61^.

SEM was used to determine if the effect of the tested materials against the ticks was related to potential structural changes. SEM analysis in this study showed irregular spots on the sensilla bases of all treated ticks; some aeropyles were blocked by secretions, and the anal groove region displayed cuticular disorders filled with irregular spots. These observations align with Agwunobi et al.^62^, where SEM images of EO-treated ticks revealed a disjointed sensilla base from the sockets, cuticular cracks, and blocked aeropyles. Additionally, the extract from *Eupatorium adenophorum* damaged the cuticular and antennal sensilla of mustard aphids *Lipathis erysimi*^63^, causing the sensilla base to detach from its socket. In general, arthropod sensilla are responsible for olfaction, which is vital for feeding behavior and response to environmental stimuli. The sensory function of cuticular sensilla is essential for the survival and normal physiology of ticks. Based on this, the ultrastructural changes observed in the cuticular sensilla of *R. sanguineus* adults suggest that the tested materials may induce severe morphophysiological disturbances, potentially leading to tick mortality. Previous reports have documented the blockage of spiracular aeropyles by liquid-like secretions, and cuticular alterations^64^. One possible reason is that the hydrophobic properties of EO disrupt the cuticular wax layer and block the spiracles of the parasite, causing mortality through desiccation or suffocation^64^.

## Conclusion

Our study demonstrated the potential of *T. vulgaris* essential oil and its nano-formulations as acaricidal agents against unfed adults of *R. sanguineus*. Additionally, these findings extend current knowledge by demonstrating morpho-histological changes and suggesting that nano-biotechnology can enhance the performance of botanical acaricides. However, the in vitro design may not fully replicate field conditions, and the high concentrations required for adult mortality may limit large-scale application. Although mortality increased over time, which may be critical for long-term effect, the delayed effect may limit their suitability in situations where rapid action is critical for tick and tick-borne disease management. Further studies are essential to evaluate safety, and environmental impact under practical conditions.

## Supplementary Information

Below is the link to the electronic supplementary material.

Supplementary Material 1

## Data Availability

All data generated or analyzed during this study are either included in this published (or available from the corresponding author upon reasonable request).
