# Supplementary material for: Efficacy of thyme oil and nano-formulated derivatives against Rhipicephalus sanguineus sensu lato (Acari: Ixodidae)
Source: Sci Rep. 2026 Feb 18;16:7384. doi: 10.1038/s41598-026-37451-9 (PMC12923577; doi:10.1038/s41598-026-37451-9)
Supplement: Supplementary file 1 — Supplementary Material 1 [file 41598_2026_37451_MOESM1_ESM.docx]

**Figures**


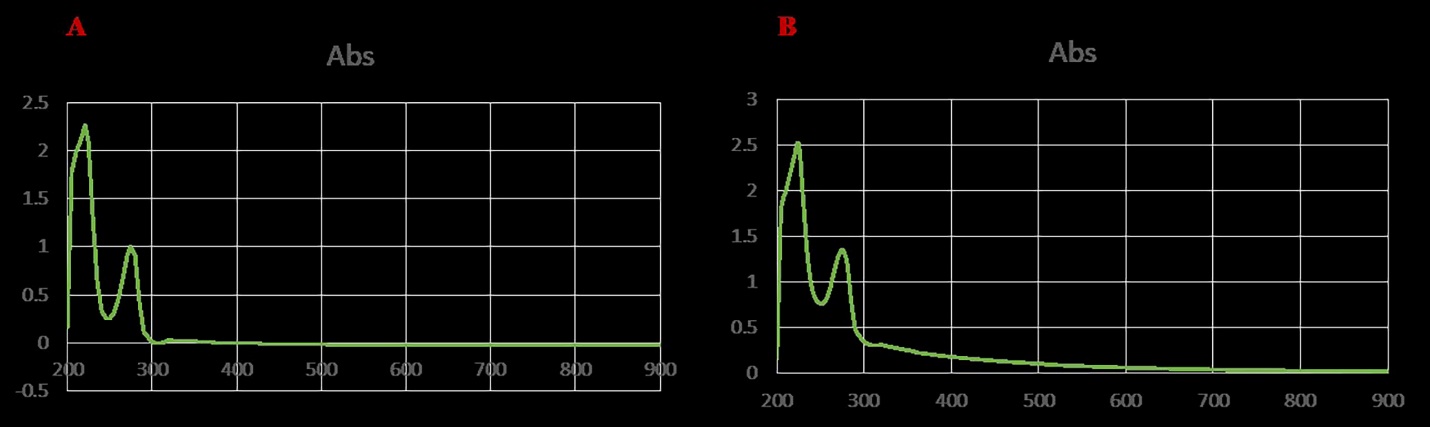


**Figure S1.** Spectral peak of known concentration in UV-Vis **A** thyme oil nano-emulsion (TNE), **B** thyme nano-emulsion containing silver (TNE – AgNPs).


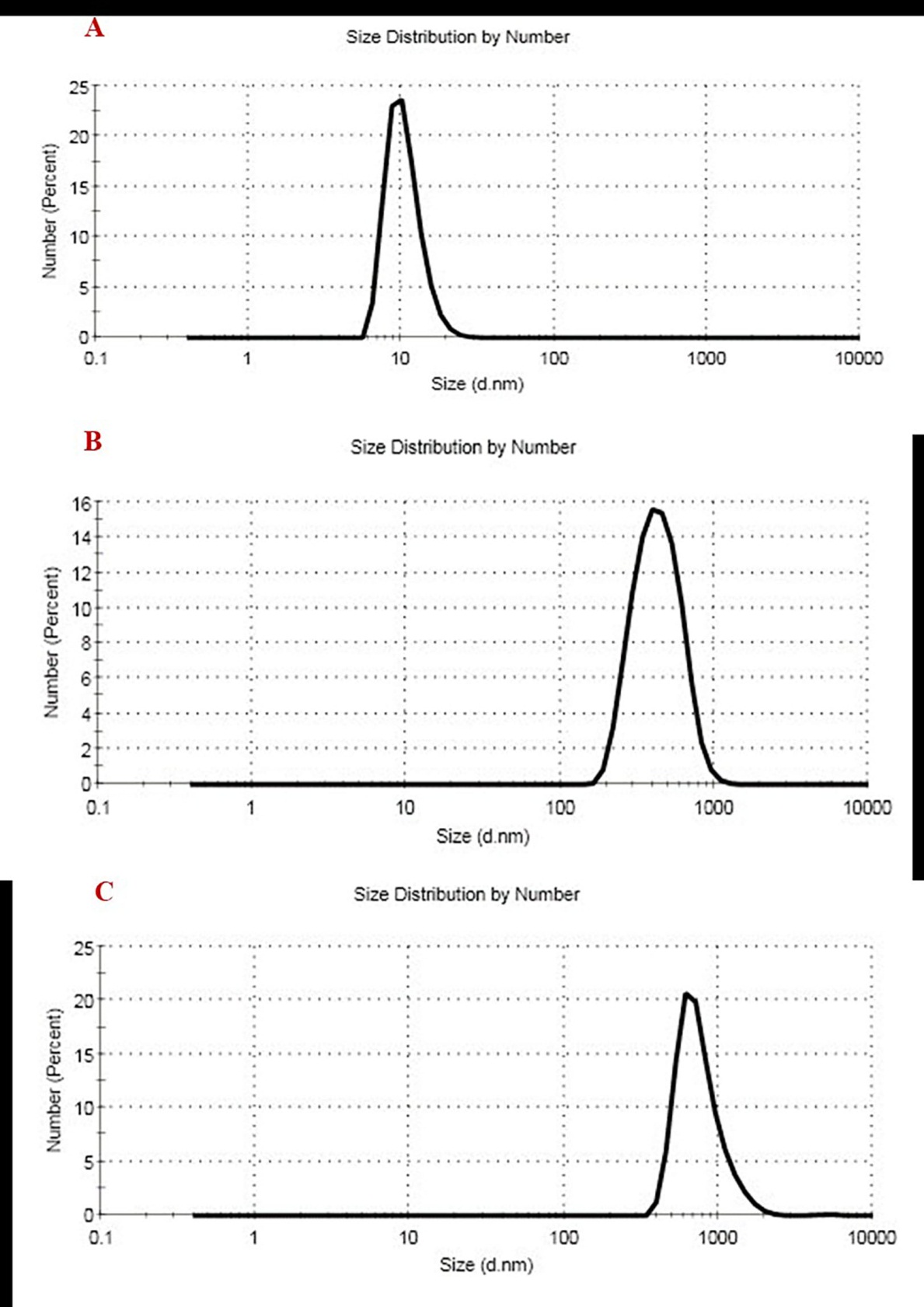


**Figure S2.** Size distribution by number for **A**. silver nanoparticle (AgNPs), **B**. thyme nano-emulsion (TNE), **C**. thyme nano-emulsion containing silver nanoparticles (TNE – AgNPs)
